# Jakinibs of All Trades: Inhibiting Cytokine Signaling in Immune-Mediated Pathologies

**DOI:** 10.3390/ph15010048

**Published:** 2021-12-30

**Authors:** Madison Alexander, Yiming Luo, Giorgio Raimondi, John J. O’Shea, Massimo Gadina

**Affiliations:** 1 Translational Immunology Section, National Institute of Arthritis, Musculoskeletal, and Skin Diseases, National Institutes of Health, 10 Center Drive, Building 10 Room 10C211, Bethesda, MD 20892, USA; madie.alexander@nih.gov; 2 Vasculitis Translational Research Program, Systemic Autoimmunity Branch, National Institute of Arthritis, Musculoskeletal, and Skin Diseases, National Institutes of Health, 9000 Rockville Pike, Bethesda, MD 20892, USA; yiming.luo@nih.gov; 3 Vascularized Composite Allotransplantation Laboratory, Department of Plastic and Reconstructive Surgery, Johns Hopkins School of Medicine, 720 Rutland Ave., Ross Research Building, Suite 755A, Baltimore, MD 21205, USA; g.raimondi@jhmi.edu; 4 Molecular Immunology and Inflammation Branch, National Institute of Arthritis, Musculoskeletal, and Skin Diseases, National Institutes of Health, 10 Center Drive, Building 10 Room 13C103C, Bethesda, MD 20892, USA; osheaj@arb.niams.nih.gov

**Keywords:** JAK, cytokines, inflammation, autoimmune diseases, COVID-19

## Abstract

Over the last 25 years, inhibition of Janus kinases (JAKs) has been pursued as a modality for treating various immune and inflammatory disorders. While the clinical development of JAK inhibitors (jakinibs) began with the investigation of their use in allogeneic transplantation, their widest successful application came in autoimmune and allergic diseases. Multiple molecules have now been approved for diseases ranging from rheumatoid and juvenile arthritis to ulcerative colitis, atopic dermatitis, graft-versus-host-disease (GVHD) and other inflammatory pathologies in 80 countries around the world. Moreover, two jakinibs have also shown surprising efficacy in the treatment of hospitalized coronavirus disease-19 (COVID-19) patients, indicating additional roles for jakinibs in infectious diseases, cytokine storms and other hyperinflammatory syndromes. Jakinibs, as a class of pharmaceutics, continue to expand in clinical applications and with the development of more selective JAK-targeting and organ-selective delivery. Importantly, jakinib safety and pharmacokinetics have been investigated alongside clinical development, further cementing the potential benefits and limits of jakinib use. This review covers jakinibs that are approved or are under late phase investigation, focusing on clinical applications, pharmacokinetic and safety profiles, and future opportunities and challenges.

## 1. Introduction

Cytokines are soluble factors that serve critical roles in intercellular immune communication and response. While cytokines ordinarily affect host defense and maintain immune homeostasis, aberrant cytokine production results in dysfunctional immune responses and immune-related pathologies. These include autoimmune, rheumatologic, allergic and inflammatory diseases, as well as disorders, such as cytokine release syndrome (CRS).

Advances in understanding cytokine biology were quickly recognized as pharmacologic opportunities; indeed, cytokines came to be employed as both treatments and drug targets. The term biologics is used to denote products encoding cytokines to treat hematologic, oncologic and infectious diseases, as well as monoclonal antibodies (mAbs) and recombinant receptors that target cytokines and cytokine receptors [1]. Biologics have been extremely successful drugs for the treatment of rheumatologic diseases, allergic pathologies and gastrointestinal conditions, including rheumatoid arthritis (RA) and other arthropathies, atopic dermatitis (AD) and inflammatory bowel disease (IBD) [2,3]. Biologics that target molecules, including tumor necrosis factor (TNF), interferons (IFNs), granulocyte-macrophage colony-stimulating factor (GM-CSF), interleukin (IL)-1, IL-4/IL-13, IL-5, IL-6, IL-17, IL-23, and IL-31, have been shown to be safe and efficacious [4,5]. Not only have these therapeutics revolutionized treatment, but they have vastly increased the field’s understanding of how cytokines drive many diseases.

Fifty-seven cytokines are known to signal via the class of receptors designated type I or type II cytokine receptors. Type I receptors, also known as hemopoietin receptors, are the cognate receptor family for the 4 α-helical cytokines which include many interleukins and some growth factors. Type II receptors are utilized by IFNs and IL-10-related cytokines. Both Type I and II receptors are structurally similar, contain an intracellular domain that binds the family of cytosolic tyrosine kinases called Janus kinases (JAKs) and are dependent on JAKs for effective signaling [6]. The JAK family includes JAK1, JAK2, JAK3 and tyrosine kinase 2 (TYK2). Binding of a cytokine to its cognate type I or II receptor induces a conformational change, leading to the activation of JAK catalytic function, which then phosphorylates tyrosine residues of the receptor’s intracellular domain. Receptor subunits that form heterodimers recruit different JAKs, whereas receptors that form homodimers recruit JAK2 to both subunits. The phosphorylation of receptor subunits allows for the recruitment of signal molecules, including latent cystolic transcription factors known as Signal Transducers and Activator of Transcription (STATs); upon recruitment, STATs are also phosphorylated by the activated JAKs. The activated STATs dimerize and translocate to the nucleus to regulate target gene expression [7].

### Establishment of JAK Criticality

The essentiality of the JAK/STAT pathway to cytokine signaling was first elegantly established using a series of mutant cell lines [8]. In vivo criticality of JAKs in cytokine signaling and immune cell development was established when patients with severe combined immunodeficiency (SCID) due to JAK3 mutations were identified [9]. These findings were later supported by Jak3 knockout mouse lines, which displayed a phenotype that largely overlapped with human SCID.

JAK1 is required in the signaling pathways of all type II cytokine receptors, cytokines that use γc, and cytokines that use glycoprotein 130, hence the severe phenotype associated with the absence of JAK1. Notably, JAK1 biallelic loss of function (LOF) mutations in humans are associated with primary immunodeficiency, mycobacterial infections, and developmental delay [10]. Conversely, JAK1 gain of function (GOF) mutations have been reported to lead to multiple system immune disease [1,11]. Genome-wide association studies (GWAS) in patients with juvenile idiopathic arthritis found an association with polymorphisms in JAK1 [12]. Jak1 germline deletion is lethal, and Jak1^-/-^ mice die perinatally [6]. Nkp46-Cre-mediated conditional deletion of Jak1 resulted in depletion of natural killer (NK) and type 1 innate lymphocyte cell populations, in addition to impaired NK development. Further, Jak1 deficiency impaired NK cell anti-tumor surveillance and activity, indicating that Jak1 is necessary for optimal innate immune function [13].

In contrast, when Jak2 was knocked out in NKp46^+^ cells, NK survival and development were not significantly affected, indicating that Jak1 is necessary and Jak2 is dispensable for NK development and survival. However, Jak2 germline deletion in mice results in impaired hematopoiesis and embryonic lethality due to JAK2′s critical role in erythropoietin signaling. Jak2 deletion in infant mice also triggers a rapid loss of hematopoietic stem cells and progenitors, leading to bone marrow failure and lethality. Notably, conditional and selective deletion of Jak2 in platelets severely affects thrombopoiesis, granulopoiesis and monocytopoiesis or leads to thrombocytosis in older mice. Conditional and selective deletion of Jak2 in megakaryocytes results in increased circulating thrombopoietin in older mice [1]. These abnormalities may be relevant in terms of the adverse events of jakinibs discussed below. Deletion of Jak2 has also been shown to impact mammary gland development and function—due to its role in prolactin signaling. Delayed puberty and impaired fertility have been observed when Jak2 is deleted in gonadotropin-releasing neurons [6]. Additionally, JAK2 GOF mutations have been identified in patients with myeloproliferative neoplasms [14].

Human TYK2 mutations similarly result in increased susceptibility to bacterial, viral, and fungal infections [15]. TYK2 deficiency in humans is also associated with altered IL-6 signaling and allergic disease, AD, as well as increased levels of immunoglobulin (Ig) E [1]. Furthermore, meta-analysis of GWAS demonstrated that some TYK2 variants are linked with autoimmune diseases, including systemic lupus erythematosus (SLE), type 1 diabetes, IBD, psoriasis, multiple sclerosis (MS), systemic sclerosis, and primary biliary cirrhosis, as well as ulcerative colitis (UC), inflammatory myopathies and Crohn’s disease (CD) [1,16]. Other TYK2 variants, however, have been found to be protective in MS, RA, psoriasis and SLE [15,17,18].

Tyk2 null mice have poor IFN and IL-12 responses, leading to exacerbated bacterial, viral, and fungal infections [19]. In a murine model of type II collagen antibody-induced arthritis, Tyk2 deficiency was associated with reduced arthritis, inflammatory cell infiltration, and destruction of articular cartilage. This was further associated with reduced production of Th1- and Th17-related and proinflammatory cytokines, such as IFNγ, IL-6, IL-1β, TNFα, IFNβ and IL-17 [20]. Similarly, in a murine model of MS, such as experimental autoimmune encephalomyelitis (EAE), Tyk2 deficiency was associated with reduced neuroinflammation [21].

Given the importance of the JAK/STAT pathway in homeostasis and immune functions, it is unsurprising that the pathway is strongly linked to cancer. Somatic mutations that activate the JAK/STAT pathway are prevalent in many cancers, as well as constitutive activation of the pathway due to upstream GOF mutations and autocrine/paracrine production of cytokines. Constitutive activation of all JAKs is linked to hematological and solid malignancies.

## 2. Jakinib Development and Approvals

The identification of JAKs’ criticality for in vivo cytokine signaling, supported by multiple lines of genetic evidence, presented JAKs as an attractive drug target. In fact, these discoveries directly led to the generation of JAK inhibitors (jakinibs), small molecules that interfere with JAKs’ enzymatic activity. The clinical development of jakinibs has shown that this class of immunomodulatory drugs is an effective alternative to biologics to target cytokines, especially since not all patients respond adequately to biologics-based treatments [1,22]. Currently, ten jakinibs are approved for humans and one is approved for canine allergic dermatitis (summarized in Table 1). The successes and shortcomings of jakinibs, since the first United States (US) Food and Drug Administration (FDA) approval in 2011, have led to the development of more selective jakinibs as well as jakinibs with multiple targets.

Early jakinib development was considered for the treatment of allogeneic transplants, as cytokines are an integral component of complex immune activation for transplanted organ rejection [22].

The first jakinib to be approved by a regulatory agency was the JAK1/2 inhibitor, ruxolitinib. Developed as a small molecule adenosine triphosphate (ATP)-competitive inhibitor, ruxolitinib targets the active conformation of the JH1 tyrosine kinase domain of JAKs [2]. Ruxolitinib was first indicated for the treatment of MPNs, including polycythemia vera, and then later approved for graft versus host disease (GVHD). More recently, ruxolitinib was found to have a superior overall response rate, durable response rate, symptom response, and failure-free survival in patients with acute GVHD when compared to other commonly used agents [24,35].

Following the positive results of the REACH-2 trial, which investigated ruxolitinib for the treatment of acute steroid refractory GVHD (SR-GVHD), ruxolitinib was quickly approved for acute SR-GVHD treatment [22,36]. Notably, in the past 30 years, no other drugs have been approved for first- or second-line treatment for acute SR-GVHD. Ruxolitinib is currently under investigation for the treatment of pediatric moderate- and severe-chronic GVHD following allogenic stem cell transplant (NCT03774082).

Additionally, ruxolitinib is used in clinical trials as a potential treatment for a variety of hematological conditions, including hemophagocytic lymphohistiocytosis (NCT04551131) and hypereosinophilic syndrome or primary eosinophilic disorders (NCT03801434). Ruxolitinib is also under investigation as an addition to anti-retroviral therapy in people living with human immunodeficiency virus to reduce inflammation (NCT02475655). The addition of ruxolitinib to anti-retroviral therapy (ART) in healthy individuals with suppressed viral loads was well tolerated and significantly decreased measures of immune activation and increased cell survival, indicating a potential utility in this setting [37].

A deuterated version of ruxolitinib, CTP-543, has been granted FDA Breakthrough Therapy and Fast Track designations by the FDA and has shown efficacy in phase I and II trials for the treatment of moderate-to-severe alopecia areata (AA). Two phase III trials are currently ongoing (NCT04518995, NCT04797650) [38].

Tofacitinib, a JAK1/JAK2/JAK3 inhibitor, was first used for the prevention of allograft rejection. However, this indication was later abandoned because the high dosage, used in conjunction with other agents, led to excessive immunosuppression [39]. Despite early setbacks, tofacitinib underwent preclinical testing in a broad range of models of diseases, including arthritis, allergy, GVHD, psoriasis and lupus [1,40]. Since 2012, tofacitinib has gained regulatory approval for four indications: RA, adult and juvenile psoriatic arthritis (PsA), polyarticular juvenile idiopathic arthritis (JIA), and UC [23]. Tofacitinib acts on the highly conserved JAK1/JAK2/JAK3 JH1 tyrosine kinase domain via ATP-competitive inhibition [2]. In a phase III trial, tofacitinib demonstrated greater efficacy than placebo in the treatment of ankylosing spondylitis (AS) (NCT03502616). Additionally, in a recently completed phase I trial evaluating tofacitinib in SLE patients (NCT02535689), tofacitinib demonstrated efficacy and tolerability, improved SLE-associated cardiometabolic and immunologic measures, improved arterial stiffness and vasorelaxation, and reduced type I IFN signatures, low-density granulocytes, and circulating neutrophil extracellular traps. Notably, patients treated with tofacitinib with the STAT4 risk allele, known to be associated with SLE, RA and other disorders, exhibited significant reduction in neutrophil netosis and improved vascular abnormalities [41]. Additional clinical trials are ongoing for tofacitinib in other autoimmune and inflammatory conditions, including cutaneous lupus (NCT03288324), Sjogren’s syndrome (NCT04496960) and inflammatory eye disease (NCT03580343).

Recently, tofacitinib has demonstrated superiority to placebo as a treatment for hospitalized, COVID-19-pneumonia patients. Patients from 15 sites were randomized to tofacitinib or placebo along with local standard of care, including use of glucocorticoids, antibiotics, anticoagulants, and antiviral agents. Tofacitinib treatment significantly reduced the risk of death or respiratory failure over a 28-day period (NCT04469114) [42].

Tofacitinib has also recently been reconsidered as a component in new combination strategies. T lymphocytes integrate signals from the T-cell receptor (TCR) and co-stimulatory receptors to orchestrate the nuclear translocation of transcription factors necessary for T cell activation [43]. The function of co-stimulatory pathways is the foundation for ‘co-stimulation blockade’ therapies to control T-cell activation and manage transplant rejection, such as abatacept (cytotoxic T-lymphocyte associated protein 4-Ig, CTLA4-Ig) [44]. In the past decade, however, investigations have found that inflammatory response impairs the co-stimulation blockade’s effect to promote long-term transplant survival [45,46]. This is likely due to cluster of differentiation (CD) 28-independent activation of T cells via inflammatory cytokine signaling [45,46,47]. To investigate the addition of tofacitinib to improve CTLA4-Ig’s effect, combined treatment of murine heart transplant models with CTLA4-Ig and short-term daily tofacitinib have been conducted. In this model, tofacitinib had a profound synergistic effect with CTLA4-Ig in promoting long-term transplant survival, even in cases with increased inflammatory response initiated by use of ischemic grafts [48]. The protective effect of the combination of CTLA4-Ig and tofacitinib was associated with inhibition of the maturation of antigen presenting cells, inhibition of the differentiation of effector T cells, and the intra-graft accumulation of regulatory T cells [48]. A parallel study examined the possibility of limiting the systemic exposure to tofacitinib while maintaining synergism with CTLA4-Ig. Single administration of a novel injectable hydrogel containing tofacitinib crystals into a rodent model localized sustained delivery of tofacitinib exclusively to the transplant and preserved synergism with CTLA4-Ig, promoting long-term graft survival [49]. This study, in combination with another that investigated delayed administration of tofacitinib in a kidney transplant rat model, offers proof-of-principle for a different utilization of jakinibs to modulate immune responses [50]. These studies demonstrated that proper selection of jakinib combination strategies can potentially achieve highly effective control of pathological immune responses and reduce the frequency of drug administration. Translational and clinical studies are clearly necessary to define if and how these strategies can be successfully implemented in clinical practice.

Baricitinib, the third jakinib approved, is a JAK1/2 inhibitor; its development has focused on autoimmune and inflammatory conditions. Baricitinib, too, reversibly inhibits JAK1/2′s JH1 tyrosine kinase domain in the active conformation and acts as an ATP-competitive inhibitor [2,51]. Both 2 mg and 4 mg daily dosages of baricitinib are effective in treating RA, and the 4 mg daily dosage has demonstrated superior efficacy to adalimumab [25]. Currently, the 2 mg and 4 mg daily dosages are approved by the European Medicines Agency (EMA) as indicated for RA and AD, whereas the FDA has approved only the 2 mg daily dose as indicated for RA. Additionally, baricitinib has shown efficacy in a phase II trial in SLE patients. Patients with active lupus and skin or joint involvement were assigned to receive 2 mg or 4 mg of baricitinib or placebo for 24 weeks. Following the treatment period, 67% of patients receiving 4 mg baricitinib and 58% of patients receiving 2 mg baricitinib achieved the primary endpoint of rash or arthritis resolution (NCT02708095) [52]. Phase III trials, BRAVE I (NCT03616912) and BRAVE II (NCT03616964), are ongoing to evaluate the safety and efficacy of baricitinib in treating SLE patients and will be followed by a long-term extension study, SLE-BRAVE-X (NCT03843125).

Baricitinib has also been shown to be efficacious in the treatment of COVID-19 patients, both as a combination treatment and monotherapy. The Adaptive COVID-19 Treatment Trial (ACCT)-2 compared the efficacy of remdesivir plus baricitinib versus remdesivir alone in patients with moderate-to-severe COVID-19 (NCT04401579) [53]. Overall, patients in the combination arm recovered after a median of 7 days compared to 8 days in the control arm. Greater benefit was observed in patients who received supplemental oxygen or non-invasive ventilation at baseline, recovering 1 and 8 days sooner with combination therapy, respectively [54]. In contrast, less favorable effects were observed in patients that did not require oxygen or intubation. In light of the results of the ACCT-2 trial, Emergency Use Authorization (EUA) was granted by the FDA for baricitinib in combination with remdesivir to treat adults and children over 2 years old who are hospitalized due to COVID-19 and require supplemental oxygen, non-invasive or invasive mechanical ventilation, or extracorporeal membrane oxygenation (ECMO) [26,55].

Subsequently, in a phase III, randomized double-blind placebo-controlled trial ACTT-4, the combination of baricitinib and remdesivir was compared to dexamethasone and remdesivir, which showed that the two interventions were comparable (NCT04640168) [56]. The effectiveness of baricitinib in combination with dexamethasone has been evaluated against dexamethasone alone in the treatment of patients with severe COVID-19 pneumonia [57]. The study found that the baricitinib–dexamethasone combination significantly reduced 30-day mortality in COVID-19 pneumonia patients compared to dexamethasone monotherapy but did not significantly alter the risk of progression to invasive mechanical ventilation or hospital-acquired infections [57]. An additional phase III, double blind, placebo-controlled study, COV-BARRIER, was conducted to evaluate the efficacy of adding baricitinib to the standard of care to treat patients hospitalized with COVID-19 (NCT04421027). The addition of 4 mg of baricitinib to the standard of care did not achieve statistical significance in the primary endpoint—patient progression to high flow oxygen, invasive mechanical ventilation (including ECMO), or death. However, there was a significant reduction (38%) in death from any cause in all groups receiving baricitinib [58]. Based on the results from COV-BARRIER, ACTT-2, and a review of baricitinib data in RA patient populations and others, in July 2021, the FDA revised the EUA to allow for the treatment of COVID-19 patients with 4 mg of baricitinib as a monotherapy, specifically for COVID-19-positive, hospitalized adults and children over 2 years old requiring supplemental oxygen and non-invasive or invasive mechanical ventilation [26]. Unlike other jakinibs, baricitinib is proposed to have antiviral activity based on its ability to inhibit AP2-associated kinase and cyclin G kinase kinases involved in SARS-CoV2 cell entry. Because baricitinib was used in conjunction with remdesivir in initial studies, the possible anti-viral role of baricitinib was obfuscated. On the other hand, baricitinib’s lack of efficacy in reducing patient progression to oxygen-based interventions while reducing risk of mortality overall could be interpreted as a lack of baricitinib-enhanced antiviral activity but rather due to baricitinib’s anti-inflammatory functions; if tofacitinib is found to have similar efficacy in COVID-19 patients it would support this possible mechanism.

Peficitinib is a pan-jakinib approved for treating RA in Japan and South Korea and is under investigation as a treatment for RA in the US and China. In a phase IIb study including RA patients, peficitinib monotherapy-treated patients achieved significantly higher American College of Rheumatology 20% criteria (ACR20) response rates than placebo-treated patients (NCT01649999) [32]. Another global phase IIb trial evaluating peficitinib in combination with conventional synthetic DMARDs (csDMARDs) found that peficitinib treatment (100 mg and 150 mg) led to a significant increase in ACR20 response rates compared to the placebo group (NCT01565655) [59]. In a follow-up two-year study recruiting from the two phase IIb global trials, patients were converted to 100 mg. The study found that peficitinib had a stable safety profile and continued to have similar ACR20 response rates as the induction trials (NCT01711814) [60]. Two phase III trials found that both 100 mg and 150 mg of peficitinib administration over 12 weeks led to clinically meaningful improvements in RA patients’ pain, work productivity, activity, and physical function (NCT02308163, NCT02305849) [61]. Peficitinib is also under investigation for moderate-to-severe UC in the US (NCT01959282), impaired renal function in Japan (NCT02603497), and moderate-to-severe psoriasis in the US (NCT01096862). In the absence of X-ray structural studies of peficitinib binding, computer modeling indicates that peficitinib non-covalently binds to the glycine-rich loop in the ATP-binding pocket of JAK molecules to mitigate JAK signaling [62].

Delgocitinib is a pan-JAK inhibitor approved in Japan for the treatment of AD and is the first jakinib to be approved as a topical formulation. Delgocitinib inhibits JAK1/2/3 and TYK2 by way of ATP-competitive inhibition [33]. Studies in adult and pediatric AD patients have indicated that delgocitinib 0.5% ointment is a safe and efficacious option to treat AD; additional investigations of delgocitinib in AD are ongoing or have been completed in the US (NCT03725722, NCT03826901) [63,64]. Early studies of delgocitinib in patients with chronic hand eczema resulted in a significantly greater number of patients achieving clearance when treated for 8 weeks with delgocitinib compared to placebo [65]. Thus, delgocitinib is currently in phase III investigations as a treatment for moderate-to-severe chronic hand eczema (NCT04871711, NCT04872101) to be followed by a long-term extension (LTE) study (NCT04949841). A phase II trial of delgocitinib ointment for the treatment of eyebrow AA was prematurely terminated due to futility (NCT03325296). An investigation of delgocitinib was performed in the treatment of adults with discoid lupus erythematosus; however, this trial was terminated due to recruitment difficulties (NCT03958955). A proof-of-concept phase II trial of delgocitinib ointment twice daily (BID) to treat mild-to-moderate inverse psoriasis was completed (NCT02695940), but the results have not yet been published.

Ocalacitinib is a JAK1/2/3 inhibitor approved for use in canines to treat allergic dermatitis. In recent studies, the use of oclacitinib was associated with a reduction in antibacterial courses compared to the use of other topical pruritis medications. Furthermore, oclacitinib demonstrated similar efficacy when compared to the use of glucocorticoids in canines and has been shown to be an improvement over the use of prednisone in canines [34]. Off-label use of oclacitinib to treat non-flea, nonfood-induced hypersensitivity dermatitis in felines and intractable Andean bear alopecia syndrome indicate the potential to expand oclacitinib to other animal species and disorders [66,67].

Following the development of these “first-generation” JAK-specific compounds, several new molecules have been developed with increased selectivity toward a specific JAK. Upadacitinib, filgotinib, abrocitinib, itacitinib and SHR0302 are reported to have a degree of selectivity toward JAK1.

Due to the high level of conservation between JAK ATP-binding pockets, upadacitinib was developed with structural differences in mind rather than differences in sequence. Thus, upadacitinib is thought to achieve JAK1 selective competitive inhibition by non-covalently interacting with the conserved glycine-rich loop in the JAK ATP-binding pocket, which is available in the “closed” conformation of JAK1 but not in the other JAKs [68]. The 15 mg once daily (QD) dose of upadacitinib, having shown superiority to adalimumab in treating RA, was approved by the FDA, making upadacitinib the first ‘JAK1-selective’ jakinib to be approved [27]. Upadacitinib has also shown superiority to abatacept in the treatment of patients with RA refractory to biologic DMARDs (bDMARDs) [69]. Upadacitinib more broadly modulated the inflammatory mediators pertinent to refractory RA pathogenesis and did so faster than abatacept. The improvement in Disease Activity Score 28 using C-reactive protein (DAS28-CRP) was also more profound in upadacitinib-treated patients than in those treated with abatacept. Interestingly, there was overlap in the mediators altered by both abatacept and upadacitinib, suggesting an overlap in these drug mechanisms [69]. Recently, long-term (60 weeks) upadacitinib treatment in RA patients, as a monotherapy or in combination with csDMARDs, was shown to improve patient-reported disease activity, pain, and physical function [70]. A recent analysis of a 3-year LTE study of upadacitinib and adalimumub on a background of methotrexate in RA patients (SELECT-COMPARE) showed that upadacitinib maintained higher levels of clinical response than adalimumab [71].

Upadacitinib has also been approved for AS by the EMA, while the extension of this indication is still under review by the FDA, primarily related to concerns regarding adverse events. Upadacitinib was approved by the EMA for the treatment of AD in adolescents and adults with or without the use of topical corticosteroids [28]. Most recently, the FDA approved 15 mg of upadacitinib as a treatment for PsA [72]. Two phase III trials have demonstrated that in PsA patients, upadacitinib has similar efficacy and safety profiles as a monotherapy or as a combination therapy with non-DMARDs and both the 15 mg and 30 mg daily dose led to improvement across endpoints (NCT03104400, NCT03104374) [73,74,75]. Upadacitinib was also efficacious in the treatment of PsA symptoms in patients that were previously classified as inadequate responders to one or more bDMARDs, and this effect was consistent irrespective of the number of previously utilized bDMARDs utilized (NCT03104374) [76]. Post-analysis additionally showed that upadacitinib was an effective treatment for active PsA, regardless of the severity of psoriasis at baseline (NCT03104400, NCT03104374) [77]. 15 mg QD of upadacitinib has also shown to be an effective means to reduce pain in AS patients with inadequate responses to non-steroidal anti-inflammatory drugs (NSAIDs) or that have NSAID contraindications. Additionally, the reduction of pain was sustained over the 90-week extension period (NCT03178487) [78]. Upadacitinib has demonstrated efficacy and safety in recent phase III trials for AD as a monotherapy and as an addition to topical corticosteroids in adolescent and adult AD patients (NCT03569293, NCT03607422 and NCT03568318) [79]. Upadacitinib is under further late-stage investigations in phase III studies of giant cell arteritis in combination with a corticosteroid taper/non-taper regimen (NCT03725202) and Takayasu arteritis in combination with a corticosteroid taper regimen (NCT04161898).

Filgotinib is another JAK1 selective jakinib approved for RA treatment by the EMA [29]. However, the FDA requested further safety data given a concern regarding male reproductive toxicity observed in preclinical studies. Filgotinib is currently being investigated in phase II and III studies for use as a CD treatment (NCT03077412, NCT02914561, NCT02914600). Recently, filgotinib showed efficacy in a phase II/III study to treat UC as a 200 mg QD oral dose (NCT02914522). Since then, the 200 mg dosage has been approved by the EMA for the treatment of adults with moderate-to-severe UC with previous inadequate, lost, or intolerant responses to conventional therapy or bDMARDs. Filgotinib has previously been assessed in patients with PsA and was found to significantly improve patients’ health-related quality of life and the Psoriatic Arthritis Impact of Disease assessment (NCT03101670) [80,81]. In the case of AS patients previously non-responsive to NSAIDs, a filgotinib phase II trial demonstrated efficacy in improving the AS disease activity score and was well tolerated (NCT03117270) [81,82]. Filgotinib non-covalently binds to the ATP-binding pocket of JAK1 in a reportedly selective manner [62].

Abrocitinib is also a JAK1 selective inhibitor being assessed in AD patients. Phase III studies with JADE Mono 1, JADE Mono 2 and JADE Compare on abrocitinib’s efficacy and safety in patients with AD have demonstrated positive results, as both 100 mg and 200 mg doses saw significant improvement in AD signs/symptoms (NCT03349060, NCT03575871, NCT03720470) [83]. Abrocitinib was recently approved by the United Kingdom (UK) Medicines and Healthcare Products Regulatory Agency (MHRA) for treatment of moderate-to-severe AD in adults and adolescents 12 years and older. Abrocitinib has also been recommended for approval by the EMA for the treatment of adults with moderate to severe AD and was granted priority review by the FDA.

Itacitinib was initially developed for acute GVHD but failed to meet the primary endpoint of its pivotal phase III trial (NCT03139604). Nonetheless, itacitinib is undergoing clinical trials to explore other indications, including asthma (NCT04129931), bronchiolitis obliterans syndrome (NCT03978637), and myelodysplastic/myeloproliferative neoplasms (MDS/MPN) overlap syndrome (NCT04061421).

SHR0302 is yet another selective JAK1 inhibitor that is in early phase clinical trials for a variety of indications, including CD (NCT03677648), RA (NCT02892370, NCT02665910, NCT02423538), AS (NCT04481139) and AA (NCT04346316). Pre-clinical studies of SHR0302 in murine models and primary cell lines from patients with acute GVHD demonstrate the potential utility of SHR0302 as treatment for acute GVHD [84]. Furthermore, in phase II trials in adults with moderate-to-severe AD, SHR0302 (administered at 8 mg and 4 mg doses) met primary and secondary endpoints with a favorable safety profile (NCT04162899). Additional late-stage trials to evaluate SHR0302 are ongoing in patients with AD (NCT04717310), UC (NCT03675477) and vitiligo (NCT04774809). Although final data has yet to be published, the sponsoring company has released positive results for SHR0302 in patients with mild-to-moderate AD, AA, and AD [85,86,87].

**Figure 1:**
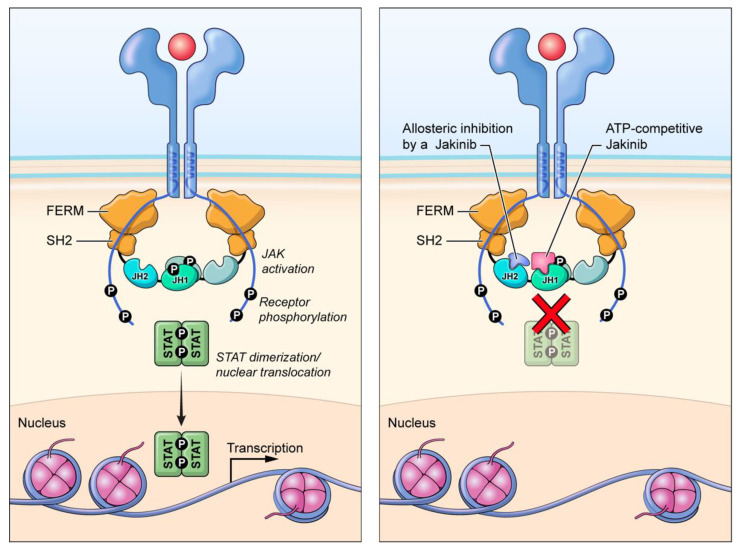
Alternate mechanisms of JAK inhibition. Deucravacitinib inhibits TYK2 activation by interacting with the pseudokinase (JH2) domain (allosteric inhibition), while first-generation jakinibs inhibit enzymatic activity by binding to the ATP-binding (JH1) domain (competitive inhibition).

As first-generation jakinibs utilize ATP-binding pocket competition to inhibit JAK activity, some “next/second-generation” jakinib development has focused on other possible mechanisms of JAK inhibition to diminish off-target effects. Deucravacitinib (BMS-986165) is a selective TYK2 inhibitor that inhibits enzyme activity via an allosteric mechanism by binding the regulatory pseudokinase domain (Figure 1) [88]. Deucravacitinib has achieved superiority over placebo and apremilast in treating moderate-to-severe plaque psoriasis in the primary endpoints and many of the secondary endpoints in two pivotal phase III trials, POETYK PSO-1 and POETYK PSO-2 (NCT03624127, NCT03611751) [89,90]. Deucravacitinib is under evaluation as a psoriasis treatment in three additional phase III trials: POETYK PSO-3, POETYK PSO-4 and POETYK PSO-LTE (NCT04167462, NCT03924427, NCT04036435). Deucravacitinib is also being studied in CD (NCT03599622), UC (NCT03934216) and SLE (NCT03252587) patients. In PsA patients, deucravacitinib has shown efficacy in a phase II trial in reducing musculoskeletal manifestations (NCT03881059) [91]. Preliminary results from a phase II study evaluating deucravacitinib in patients with PsA demonstrated that more patients taking deucravacitinib achieved ACR20 compared to the placebo group and deucravacitinib achieved secondary endpoint measures (NCT03881059) [92].

Brepocitinib (PF-06700841) is an ATP-competitive, selective JAK1/TYK2 inhibitor [2]. Currently, brepocitinib is in phase II trials for the treatment of psoriasis (NCT03850483) and AD (NCT03903822) as a topical cream, and orally for PsA (NCT03963401), UC (NCT02958865), CD (NCT03395184), vitiligo (NCT03715829), SLE (NCT03845517), cicatricial alopecia (NCT05076006), and hidradenitis suppurativa (HS, NCT04092452). In a phase II/III trial for AA, brepocitinib treatment led to clinically significant hair regrowth, as assessed by the Severity of Alopecia Tool (SALT), after 24 weeks of treatment in patients with ≥50% scalp hair loss (NCT02974868) [93].

In addition to efforts to improve jakinib selectivity, other jakinibs inhibit multiple signaling pathways. One such inhibitor is gusacitinib, a pan-JAK/ spleen tyrosine kinase (SYK) inhibitor that is being developed for AD (NCT03531957) and chronic hand eczema (NCT03728504). Cerdulatinib is an ATP-competitive, reversible JAK1/JAK2/SYK inhibitor that has shown tolerability and clinical activity in follicular lymphoma and T-cell lymphomas (NCT04021082, NCT01994382), although trials are currently on hold [94,95,96]. A topical formulation of cerdulatinib is also under development for vitiligo (NCT04103060).

Fedratinib is a JAK2/FMS-like tyrosine kinase 3 (FLT3) inhibitor that received FDA approval for the treatment of myelofibrosis. Momelotinib is a JAK1/JAK2/activin receptor type-1 (ACVR1) ATP-competitive inhibitor that is being investigated in patients with myelofibrosis who have failed previous jakinib therapy (NCT01969838) [97]. Additionally, momelotinib is under investigation as a treatment for primary myelofibrosis, post-polycythemia vera, and post-essential thrombocythemia myelofibrosis patients that are anemic or thrombocytopenic following ruxolitinib treatment (NCT02101268).

Pacritinib is a novel “multi-target” agent, developed for myelofibrosis, which inhibits JAK2/FLT3/IL-1 receptor associated kinase (IRAK1)/colony stimulating factor 1 receptor (CFS1R). Pacritinib preferentially binds to JAK2′s activated form to act as an ATP-competitive inhibitor [98,99]. In a phase II trial, pacritinib demonstrated the potential advantage of treating severe thrombocytopenia in patients with primary or secondary myelofibrosis due to its multiple-target-based design (NCT01773187) [100].

Ritlecitinib (PF-06651600) is an oral, JAK3/Tec kinase inhibitor currently under investigation in a phase III trial for the treatment of AA (NCT03732807) and in phase II trials for vitiligo (NCT03715829), UC (NCT02958865), and CD (NCT03395184). Ritlecitinib met its primary endpoints for scalp hair loss and achieved hair regrowth in a 24-week AA study (NCT03732807) [93]. The extension study of ritlecitinib in AA patients is ongoing (NCT04006457) [101]. Ritlecitinib achieves JAK3 selectivity by covalently and irreversibly binding to a non-conserved cysteine in the JAK3 JH1 ATP-binding pocket [2].

In addition to expanded target pathways, second-generation jakinibs include non-absorbable molecules that seek to reduce systemic exposures and enhance local responses. Izencitinib (TD-1473) and OST-122 are two gut-selective jakinibs. Izencitinib is a reversible pan-JAK inhibitor that has been shown to have high intestinal-to-plasma drug exposure ratios in pre-clinical and early clinical studies (NCT02818686) [102]. Currently, izencitinib is in phase II and phase III trials to investigate its efficacy in the treatment of IBD, including both UC (NCT03758443) and CD (NCT03635112). OST-122 is a gut-selective “multi-target” agent that inhibits JAK3/TYK2/AMPK-related protein kinase 5 (ARK5) and is currently in a phase II trial for the treatment of UC (NCT04353791).

TD-8236 is an inhaled pan-JAK inhibitor in trials for the treatment of moderate-to-severe asthma. Reports from a phase I/II trial show inhibition of fractional exhaled nitric oxide (FeNo) levels, suggesting engagement of the IL-13 pathway, which drives eosinophilic-mediated disease (NCT03652038) [103]. A reduction in STAT phosphorylation in the sputum of asthmatic patients was also observed upon TD-8236 administration; however, TD-8236 did not meet its primary endpoint in a phase IIa lung allergen challenge (NCT04150341) [103]. The potential utility of inhaled jakinibs is also being studied in COVID-19 with a different agent, nezulcitinib (NCT04402866) [104].

A summary of jakinib clinical trials can be found in Table 2.

## 3. Safety of Jakinibs

In the twenty years since the first approval of jakinibs, their safety profiles have been studied extensively and we now have considerable data relating to this topic. Still, there are many unanswered questions. Given that jakinibs block numerous cytokines that are involved in many aspects of host defense, hematopoiesis, metabolism, cell growth, and cell differentiation, it is not surprising that jakinibs can have multi-systemic impacts. Indeed, it can be argued that it was a surprise just how safe these agents have shown to be when used at doses that provide reasonable efficacy. Nonetheless, the hope of second generation jakinibs with putative increased selectivity is to preserve efficacy and reduce adverse events. Here, we will discuss the major side effects of jakinibs, comparing first- and second-generation jakinibs.

### 3.1. Infections

As immunosuppressive agents, appropriately dosed jakinibs are associated with a risk for infection that is generally comparable to bDMARDs. However, patients taking jakinibs are at greater risk for herpes zoster infection or reactivation when compared to patients taking DMARDs. Of more serious consequence, first-generation jakinibs have also been seen to increase patient risk for opportunistic infection and serious infections, such as reactivation of *Mycobacterium tuberculosis*, *Pneumocystis jirovecii* pneumonia, oral/esophageal candidiasis, Hepatitis C, varicella-zoster, disseminated or serious herpes zoster, and BK nephropathy [60,105,106].

Such complications altered the course of early jakinib development for GVHD. When tofacitinib was first tested in a phase I trial at 5 mg, 15 mg, and 30 mg BID in stable renal transplant patients and co-administered with calcineurin inhibitors at lower doses, the safety and tolerability profiles were deemed acceptable [40,107]. However, in the follow-up phase II trial comparing 15 mg and 30 mg of tofacitinib to tacrolimus, high doses of tofacitinib increased patients’ risk for complications from immunodeficiency, notably for cytomegalovirus infections and BK nephropathy [39]. Additionally, the combination of tofacitinib and mycophenolate (MPA) in transplant recipients has been associated with a persistent risk of serious infections. Reconsideration of the data from these studies and the results of an LTE trial have highlighted the importance of and need for controlled tofacitinib dosing and careful evaluation of drug combination strategies [107,108]. However, numerous studies in transplant settings have demonstrated that not only are jakinibs efficacious, but the rates of jakinib-related infections are similar to biologics-related infection rates in transplant patients. This suggests that in the context of solid organ transplantation management, jakinibs require a tailored application.

Akin to the broader class of jakinibs, the most common side effects of putative JAK1-selective inhibitors, filgotinib and upadacitinib, are infections [109,110]. In the case of upadacitinib in RA patients, the risk of overall infection was similar to that of the TNF inhibitor adalimumab. In patients with AD, upadacitinib increased the risk for herpes zoster infection when compared to the standard treatment of dupilumab, an IL-4 and IL-13 inhibitor (NCT037383970) [111]. Similarly, in RA patients, upadacitinib, on a background of methotrexate, increased the risk of herpes zoster infection compared to adalimumab [112].

A recent meta-analysis compared the safety data of patients prescribed 5 mg tofacitinib, 4 mg baricitinib, 15 mg upadacitinib, 100 mg filgotinib, or 200 mg filgotinib. This study found no significant differences in the incidence of severe AEs and herpes zoster infections between the drug groups and dosages analyzed. Ranking probabilities predicted that the 5 mg dosage of tofacitinib is the safest treatment (in addition to placebo), followed by 200 mg filgotinib, 100 mg filgotinib, 4 mg baricitinib, and 15 mg upadacitinib based on the non-significant differences in safety profiles identified [113]. The overall findings of this study suggest that JAK-selectivity does not significantly reduce the risk for infection compared to first-generation jakinibs in RA patients. Compared to the studies that focus on the different inhibitors on a more ‘individual’ scale, this meta-study questions if increased JAK selectivity effectively reduces the risk of AEs. In the case of infection risk in RA patients, this does not appear to be the case.

Like other jakinibs, deucravacitinib’s most common AEs are upper respiratory infections [114]. In a recent study of deucravacitinib in PsA patients, the most common upper respiratory infections were nasopharyngitis and sinusitis. No herpes zoster infections or opportunistic infections were reported. However, this study was conducted in 203 patients; thus, less common infections may not have been apparent [115]. Similarly, in a phase II trial, no psoriasis patients taking deucravacitinib experienced herpes zoster infections during the 96-day treatment period, indicating that JAK-selectivity may decrease the jakinib-associated risk of herpes zoster infection [88].

Other selective inhibitors under investigation, brepocitinib (a JAK1/TYK2 inhibitor) and ritlecitinib (a JAK3/Tec kinase inhibitor), have indicated that selectivity may reduce the risk for infection. In a phase II/III evaluated in AA patients, no cases of opportunistic infections were reported among patients taking brepocitinib or ritlecitinib [93].

### 3.2. Hematologic Effects

Another clinical measure of interest to the safety profile of jakinibs is anemia and changes in hemoglobin levels. Early safety studies of tofacitinib found that patients taking 30 mg BID had an increased incidence of anemia, leukopenia, neutropenia, lymphopenia, and thrombocytopenia compared to patients treated with 5 mg or 15 mg tofacitinib or placebo [116]. In phase IIb dose-ranging studies for tofacitinib, severe anemia (hemoglobin levels of 7–8 gm/dl or a decrease of 2–3 gm/dl from baseline) was seen in 2.8%, 4.2%, and 6.5% of patients taking 3 mg BID, 10 mg BID, and 15 mg BID, respectively [117]. Analysis of pooled phase III tofacitinib trials in patients with RA showed that although the 5 mg and 10 mg tofacitinib-treated groups had reductions in hemoglobin over a 24-month period, less than 1% of tofacitinib patients, at either dose, experienced clinically meaningful decreases in hemoglobin [118]. These investigations suggest that in RA patients, tofacitinib likely increases the risk for anemia in a dose-dependent manner. In a LTE study of baricitinib in RA patients, there were no demonstrated significant changes to platelet counts between treatment or dosing groups (2 mg and 4 mg) [119]. Analysis of eight pooled baricitinib trials found that in both baricitinib groups, patients saw small decreases in hemoglobin levels, which later returned to or exceeded the baseline level after 48–52 weeks of treatment. Across these studies, analysis revealed that 50% of baricitinib patients experienced AEs related to decreased hemoglobin levels, and 0.5% of patients discontinued treatment due to decreased hemoglobin or anemia [120]. As JAK2 inhibition is thought to be affiliated with the AEs of anemia seen in patients taking tofacitinib and baricitinib, likely due to JAK2′s role in erythropoiesis, it was suggested that upadacitinib’s purported JAK1-selectivity would reduce rates of anemia. In a phase IIb/III trial, rates of anemia similarly appeared dose-dependent, as 0%, 1.6%, and 4.9% of patients taking upadacitinib at 7.5 mg QD, 15 mg QD, and 30 mg QD, respectively, experienced anemia. A similar dose dependency was seen for neutropenia and lymphopenia as well, with the highest rates occurring in the 30 mg QD group. However, no patient discontinued the treatment because of anemia [121]. Further studies have found an association between the 15 mg dose of upadacitinib and anemia [121,122]. Overall, the rates of anemia in upadacitinib-treated patients indicate that this drug is, at best, only relatively selective for JAK1.

Supporting the role of JAK2-inhibition in the pathogenesis of anemia, a phase II study found that deucravacitinib did not significantly alter levels of lymphocytes, NK cells, neutrophils, platelets, or hemoglobin [88]. This indicates that TYK2-selective inhibition may improve the hematologic safety profiles by allowing JAK2-dependent signaling to remain intact.

Jakinibs, as a class, have also been associated with reversible cytopenia (particularly of lymphocytes, NK cells, neutrophils, and platelets), hyperlipidemia, mild elevation of transaminase, creatinine, and creatinine kinase levels [119,123,124,125,126]. However, most patients with jakinib-induced laboratory changes do not appear to develop serious AEs [127]. As mentioned above, deucravacitinib did not significantly decrease lymphocyte, NK cell, neutrophil, platelet, or hemoglobin levels [115,128]. In a phase II/II trial evaluating other selective jakinibs, ritlecitinib and brepocitinib, no clinically relevant changes from baseline were identified in hematology measures; however, one ritlecitinib patient did have decreased lymphocyte counts and two brepocitinib patients had decreased neutrophil counts [93].

In summary, selective JAK targeting may indeed prove to be advantageous in preventing the multi-JAK inhibition-induced adverse effects to hematopoietic cell populations.

### 3.3. Thromboembolic Risk

Baricitinib, tofacitinib, and upadacitinib increase the risk of venous thromboembolism (VTE), deep venous thromboembolism (DVT), and pulmonary embolism in patients with RA, psoriasis, PsA, and UC. In the case of upadacitinib, DVT was rare and only seen in patients treated with 30 mg and 45 mg of upadacitinib, unlike the approved 15 mg dose in RA patients [121,122]. Jakinib-associated thromboembolic events should be considered in the larger context, as RA patients have an increased risk of thromboembolic events from baseline; thus, the role of RA versus jakinibs in thromboembolic pathogenesis requires further investigation [106]. Thromboembolic risk with jakinib use is of particular concern in the treatment of COVID-19 patients. However, unlike the increased risk in patients with RA, the ACCT-2 study and others found that no excess thromboembolic events have emerged in jakinib study arms [53,54,129,130,131]. In fact, administration of baricitinib and glucocorticoids in combination resulted in a greater decrease in D-dimer levels than glucocorticoids alone [132].

### 3.4. Impact on Lipids

Tofacitinib administration in patients with UC was not associated with significantly elevated lipids when compared to the placebo; however, the long-term study is still ongoing [133]. In contrast, in psoriasis patients treated with tofacitinib increases in triglycerides, low-density lipoprotein cholesterol (LDL-C), and high-density lipoprotein cholesterol (HDL-C) were seen; however neither the total cholesterol/HDL-C ratio nor the LDL-C/HDL-C ratio changed. This is somewhat in line with an increased risk of major adverse cardiac events (MACE) in tofacitinib patients, as higher LDL-C levels are associated with increased cardiovascular (CV) risk, while increased HDL-C levels are associated with decreased CV risk [134]. However, in an SLE-tofacitinib phase I study, tofacitinib was associated with an increase in HDL-C, whereas LDL and triglyceride levels remained unchanged [41].

In a LTE study of baricitinib safety, RA patients taking 4 mg of baricitinib saw a significant increase in HDL-C and LDL-C serum concentrations compared to those treated with placebo [119]. Meta-analysis of six baricitinib trials in RA patients also saw significant increases in LDL-C and HDL-C; when the net mean change from placebo-treated groups was adjusted for potential confounders, LDL-C levels increased slightly more on average in comparison to HDL-C levels across the pooled studies. Further, the LDL-C and HDL-C increases were indicated to be dose-responsive to increasing baricitinib dosage, yet baricitinib was not found to be associated with increased CV risk [135].

The same study also estimated the effect of other jakinibs on lipid levels in patients with RA. Tofacitinib and decernotinib, a reportedly selective JAK3 inhibitor, treatment led to greater increases in LDL-C from the placebo controls when compared to baricitinib-induced increases in LDL-C. Filgotinib was estimated to moderately induce LDL-C concentration increases and peficitinib did not significantly alter LDL-C levels compared to placebo groups. Overall, tofacitinib, decernotinib, filgotinib, and peficitinib were all estimated to induce HDL-C serum concentration increases, however to a lesser extent than the induced increases in LDL-C [135].

In a phase II/III study of brepocitinib and ritlecitinib in AA patients, no clinically relevant changes in LDL-C nor LDL-C/HDL-C ratios occurred compared to baseline. However, in the brepocitinib-treated group, a slight increase in HDL-C was reported [93]. Unlike the TYK2/JAK1-selective brepocitinib, TYK2-selective deucravacitinib did not demonstrate a significant association with dyslipidemia in the psoriasis trial, indicating the potential improvement in the safety profile of TYK2-selective targeting [88]. Further, deucravacitinib in PsA patients did not significantly increase serum lipid concentrations or mean levels of serum cholesterol [115,128].

Studies to classify the clinical impacts of jakinib-associated lipid changes in different patient populations can help to delineate the risks and benefits of jakinibs in different patients and help to better understand the relationship between jakinib-induced CV risk and lipid concentrations increases. Further, the indicated differential effects of JAK selectivity on lipid increases calls to question the viability of targeting specific JAKs with next-generation jakinibs to reduce off-target affects that may contribute to increased patient risk.

### 3.5. Cardiovascular and Malignancy Risk

The ORAL Surveillance A3921133 study (NCT02092467), a regulatory agency-required open-label randomized controlled trial (RCT), compared the safety profiles of tofacitinib and TNF inhibitors for CV and malignancy risk in RA patients. An interim analysis in 2019 revealed numerically more cases of VTE and death in those taking 10 mg BID dosage of tofacitinib. There were reportedly 19 cases of pulmonary embolism (PE) and 45 cases of death among 3884 patient-year follow-up in patients taking tofacitinib, compared to 3 cases of PE and 25 cases of death among 3982 patient-year following in patients taking TNF inhibitors as of early 2019 [136]. However, the detailed results have not yet been published. Based on this information, the FDA issued a warning about the increased risk of VTE and death for the 10 mg BID dosage of tofacitinib. In early 2021, the FDA disclosed the results of the A3921133 study.

Upon further review, the FDA mandated an expansion of the boxed warning attached to tofacitinib to include baricitinib and upadacitinib for an increased risk of CV events, including heart attack or stroke, blood clots, malignancies (excluding non-melanoma skin cancer (NMSC)), and death for those receiving 5 mg or 10 mg dosage of tofacitinib [137]. Although fewer studies have evaluated baricitinib and upadacitinib for an increased risk for these conditions, the FDA ruled that because they share a mechanism (JAK1 inhibition) and are indicated for the treatment of RA and inflammatory conditions, such as AD, they may carry the same risks to these patient populations. However, ruxolitinib and fedratinib were not included in the warning expansion, as they are not indicated for the treatment of arthritis or other inflammatory conditions.

The sponsor of the study released preliminary findings that stated a hazard ratio (HR) of 1.33 for adjudicated MACE with a 95% confidence interval (CI) between 0.91 and 1.94 (95% CI 0.91, 1.94). Further, the sponsors reported an HR of 1.48 (95% CI 1.04, 2.09) for adjudicated malignancies, excluding NMSC, for tofacitinib compared to TNF inhibitors [138]. The hazard ratio CI crossed 1.0 for MACE and crossed the pre-specified non-inferiority margin of 1.8 for both MACE and malignancies. Thus, it is inconclusive whether tofacitinib has clinically significant inferior safety profiles in CV and malignancy risk compared to TNF inhibitors. More detailed data was also released in the “Study Results” section of this study (NCT02092467) on the ClinicalTrials.gov website but has not yet been published in a peer-reviewed journal. The finding of increased VTE risk from the A3921133 study is consistent with a recent real-world study using WHO VigiBase but inconsistent with two meta-analysis pooling data from major RCTs of jakinibs [139]. Furthermore, the findings that tofacitinib increased risk for MACE and malignancies are inconsistent with a recent real-world study that compared tofacitinib to bDMARDs among patients in the US Corona RA registry [140]. Real-world evidence reported that the incidence of MACE and malignancies over 5 years for RA patients taking tofacitinib or bDMARDs was similar [140]. There is also a possibility that the discrepancies of CVevents in the A3921133 study were caused by a reduced risk of MACE in patients taking TNF inhibitors [141,142].

Further investigations have been dedicated to determining CV risk associated with tofacitinib use in comparison to TNF inhibitors. Databases of RA patients treated with tofacitinib or TNF inhibitors from ‘Optum’ Clinformatics, IBM ‘Marketscan’, and Medicare were used to model an RCT-like cohort and a real-world evidence cohort. The analysis found that in the RCT-like cohort, tofacitinib treatment increased the risk of CV compared to TNF inhibitors in RA patients with preexisting CV risk factors. Conversely, the real-world analysis did not find that tofacitinib increased CV risk in RA patients when compared to TNF inhibitors. These data were presented in abstract form and have yet to be peer-reviewed [143]. Additional results from a phase IIIb/IV study presented in abstract form identified independent risk factors for MACE in RA patients with existing CV risk and/or aged 50 years and older. The study found that incidence rates of MACE (including non-fatal MACE) and non-fatal myocardial infarction (MI) were numerically higher in tofacitinib-treated RA patients compared to those treated with TNF inhibitors. However, incidence rates for fatal MI and stroke were similar between tofacitinib- and TNF-treated patients. Hazard ratios for MACE, MI, and stroke were greater than 1.0 when tofacitinib treatment was compared to TNF inhibitor treatment. The most important CV risk factors for MACE were active smoking, current aspirin use, being 65 years and older, and being male; even a single risk factor was sufficient to non-significantly increase the incidence rate of MACE in both treatment groups. With or without these risk factors, the hazard ratios for MACE were greater than 1.0 in the tofacitinib group compared to the TNF inhibitor group [144]. These data indicate that while there are risk factors that increase the risk of CV complications independent of tofacitinib use, the incidence of MACE remains numerically higher in patients treated with tofacitinib compared to TNF inhibitors.

As in RA, patients with IBD also have an increased CV risk. Thus, concern for MACE in UC patients treated with tofacitinib led to the assessment of lipid levels and CV risk in recent UC-tofacitinib studies (NCT01465763, NCT01458951, NCT01458574, NCT01470612) [133]; however, tofacitinib treatment was not found to be associated with a significantly increased risk of MACE.

Overall, jakinib-induced CV risk and dyslipidemia require further investigation in multiple patient populations. Although these safety data do not appear to be in absolute agreement, a significantly increased risk of MACE, malignancy, and death in some patient populations treated with tofacitinib has been observed. These concerns highlight the complexities of clinical jakinib use and the potential ramifications that can come with the benefits of JAK/STAT pathway inhibition, especially in the case of pan- or multi-jakinibs.

As jakinibs continue to be developed, long-term safety studies will become increasingly important to identifying potential AEs and risk factors. In the case of baricitinib, the safety results of an analysis of nine phase III/II/Ib and one LTE were recently published [145]. The study extended to up to 9.3 years of baricitinib usage in active RA patients. Analysis identified that early safety signals of increased risk of serious infections, herpes zoster infections, and an association with increased risk of MACE were maintained over the LTE, and that in the long-term incidence rates of malignancy and DVT/PE was 0.74–1.03 and 0.3–0.5, respectively, consistent with short-term data [145]. As in the case of baricitinib use in RA patients, LTE studies remain necessary to evaluate jakinib safety, especially in the treatment of chronic diseases that are themselves associated with increased risk. Further, it remains to be investigated if JAK selectivity improves jakinib safety profiles in a similar manner across various patient populations. Importantly, the extent to which selective jakinibs improve clinical measures compared to first-generation jakinibs across patient populations may help determine if there are significant and clinically meaningful benefits of JAK selectivity.

## 4. Selectivity and Pharmacokinetics of Jakinibs

In considering the safety and efficacy of jakinibs and where jakinib development might go next, it is worth reviewing topics that are sometimes neglected in reviews: selectivity and pharmacokinetics (PK). Selectivity assessments of jakinibs are of increasing importance with the development of more selective jakinibs and attempts to delineate the mechanisms of jakinib effects versus their side effects. Like all drugs, jakinibs each have a unique pharmacologic profile. Thus, in considering mechanisms of action and selectivity in relation to efficacy, the role of PK and pharmacodynamics (PD) should also be assessed. As we increase our understanding of JAK selectivity, bettering our knowledge of jakinib pharmacokinetics will provide a more complete picture of how jakinibs behave in vivo and the effects of increasing selectivity.

With respect to selectivity, most jakinibs were identified and developed using screening assays based on inhibition of catalytic activity of recombinant JAK kinase domains. In vitro, cell-based assays have been employed to define relative JAK selectivity; the most essential information is the documentation of target engagement in relevant cell types and in humans receiving these drugs [146,147].

Peripheral blood samples are often used to assess target engagement, but this does not necessarily directly translate to drug actions in diseased tissue. Additionally, since direct measurement of JAK enzymatic activity in patient-derived cells is not really possible at this time, surrogates to measure JAK inhibition are required. Phosphorylation of STATs is a reasonable surrogate; however, the action of jakinibs is lost nearly immediately if cells are processed and the drug of interest is removed [146]. Moreover, since there are seven STATs and 57 cytokines dependent upon JAKs, it is difficult to define which assay is most informative [1]. We have a reasonable understanding of genes induced by various cytokines, yet again, there is a great deal of overlap. For all these reasons, operationally defining selectivity is anything but a simple issue.

An illustrative example comes from a recent comparison of tofacitinib, baricitinib, and upadacitinib. In an in vitro assay in which peripheral blood mononuclear cells (PBMCs) from healthy donors were incubated with clinically relevant doses (calculated as mean concentration–time profiles over 24 hours obtained from jakinibs-treated subjects) of tofacitinib, baricitinib, and upadacitinib and STATs phosphorylation was assessed by flow cytometry focused on phenotypically-gated leukocyte subpopulations. While baricitinib inhibited JAK1/3 signaling to a lesser extent than upadacitinib and tofacitinib, quite surprisingly, JAK1-selective upadacitinib was the most potent inhibitor of the JAK2-dependent cytokines IL-3 and GM-CSF, whereas tofacitinib was the most potent inhibitor of granulocyte colony stimulating factor (G-CSF), a JAK2/TYK2 dependent cytokine [146]. Tofacitinib, baricitinib, and upadacitinib all demonstrated IL-6, IFNγ, and IL-10 inhibition. More specifically, tofacitinib was the most potent inhibitor of IL-6, and upadacitinib and tofacitinib demonstrated greater inhibitory potency toward IFNγ than baricitinib [146]. As these studies were conducted using clinically relevant doses in vitro, it brings into question the purported JAK1-selectivity of upadacitinib, as it was demonstrated to be a potent inhibitor of JAK2- and JAK1/TYK2-dependent cytokines. Another example of an in vitro surrogate assay is a microfluidics assay that has been used to measure the phosphorylation of recombinant human JAK kinase domains in the presence of jakinibs [148].

Another salient example is the comparison of baricitinib, filgotinib, filgotinib’s major metabolite (GS-829845), tofacitinib, and upadacitinib using a similar experimental approach (e.g., assessing phosphorylated STAT (pSTAT) in PBMCs or whole blood after cytokine stimulation). Interestingly, the authors in this study utilized PBMCs and whole blood from healthy controls, as well as from RA patients, and demonstrated that all the jakinibs evaluated showed similar JAK1-dependent cytokine inhibitory activity. Filgotinib exhibited less inhibition of JAK2- and JAK3-dependent cytokines compared to upadacitinib, baricitinib, and tofacitinib [147]. However, the in vitro/ex vivo nature of the study presents several limitations. An additional selectivity study focused on deucravacitinib, tofacitinib, baricitinib, and upadacitinib via in vitro half maximal inhibitory concentration (IC50) analyses. At clinically relevant concentrations, deucravacitinib demonstrated selectivity for TYK2 and little inhibitory activity against JAK1/2/3-dependent cytokine stimulation. In comparison, the study found that tofacitinib, baricitinib, and upadacitinib were more potent inhibitors of JAK1/2-dependent and JAK2/2-dependent cytokine signaling [149]. In line with the above study, upadacitinib had IC50 values comparable to baricitinib in JAK2/2-dependent cytokine signaling inhibition, further suggesting that upadacitinib may have JAK2 inhibitory activity. Undoubtedly, the results of these studies are limited, as only surrogates for JAK activity were used and investigators only measured a single JAK-induced cytokine/pSTAT per JAK coupling evaluated. Jakinibs’ effects are influenced by a myriad of factors, such as the cytokine utilized for each assay, the STAT being assessed, and the cell type being analyzed. It is imperative to consider all these factors and the correlations between them when determining JAK selectivity.

Although patient studies can provide a more translatable view of JAK selectivity, they can be obscured due to patient complexity and variation, as well as the inability to directly measure selectivity in vivo. A recent example is a study of deucravacitinib in PsA patients in a phase II trial (NCT03881059). Preliminary data suggest that upon the evaluation of biomarkers and adverse effects downstream of JAK signaling, deucravacitinib did not alter serum and hematologic variables known to change with JAK1/2/3 [128]. Despite a lack of conclusivity, this data supports the TYK2-selectivity of deucravacitinib in patients.

Linking drug action with cytokines can be further complicated by alterations in cell populations. For instance, anemia certainly may implicate inhibition of JAK2, given its role in hematopoiesis; however, cytokines, such as IL-11, a glycoprotein (gp)130-using cytokine, are also involved in platelet homeostasis. Additionally, a highly specific JAK3 inhibitor would not be expected to influence INFγ induction by IL-12, which signals via TYK2 and JAK2; nonetheless, inhibiting JAK3 causes a reduction in NK cells, which are major producers of IFNγ. Similarly, cytokines are regulated in complex networks; for example, TNF and IL-1 are induced by IL-6 and as a result, jakinibs can have indirect effects on cytokines that do not signal via this pathway. These considerations are relevant to efficacy and safety. The bottom-line measurement of true selectivity in vivo is not a simple task and is not always reflective of in vivo selectivity and the resulting clinical effects.

PK studies of jakinibs have been conducted along with jakinib safety and efficacy trials, as a vital component to understanding how jakinibs perform and are metabolized and eliminated in patients. As a class, jakinibs are readily absorbed, reaching peak plasma concentrations shortly after oral administration. Similarly, jakinibs are quickly cleared, predominantly by the liver in kidneys. We have a reasonable understanding of the PKs of ruxolinitinib, tofacitinib, baricitinib, and upadacitinib and how this relates to modulating cytokine signaling. Some jakinibs primary metabolites have been reported to maintain activity, and thus must be included when evaluating PK profiles. These jakinibs’ time to peak concentration and functional half-lives are summarized in Table 3. Ruxolitinib is metabolized into eight sub-species, which are suggested to retain JAK1 and JAK2 inhibitory activity [150]. Filgotinib’s primary metabolite, GS829845, exhibits a ten-fold reduction in potency against JAK1 but maintains JAK1 selectivity (NCT01179581, NCT01419990, NCT01384422). GS829845 likely extends the absorption period and half-life of filgotinib overall.

Ruxolitinib is metabolized by cytochrome p450 enzymes (CYP); thus, co-administration of cytochrome enzyme inhibitors has been shown to alter oral clearance rates and ruxolitinib half-life [150,156]. Ruxolitinib is metabolized predominantly by CYP3A4 and CYP2C19, although CYP2C19 has a smaller role. Studies have been conducted using CYP3A4 inhibitors and CYP3A4 inducers, ketoconazole, erythromycin and rifampin, in combination with ruxolitinib [150]. Co-administration of ketoconazole increased ruxolitinib exposure and prolonged ruxolitinib’s half-life. Co-administration of erythromycin, a less potent CYP3A4 inhibitor, did not impact ruxolitinib exposure and elimination as significantly as the more potent ketoconazole. Rifampin co-administration decreased the exposure and half-life of ruxolitinib by approximately 52% and 50%, respectively [112,150]. PK modeling of the effect of co-administration of a CYP3A4 and CYP2C19 inhibitor, fluconazole, estimated that co-administration would lead to a two-fold increase in ruxolitinib’s area under the curve (AUC) [112,157]. Despite the mechanism of hepatic ruxolitinib metabolism, no clear correlation has yet been established between the degree of hepatic impairment and altered ruxolitinib exposure. Still, a decreased starting dose in patients with hepatic impairments is recommended for ruxolitinib [158].

PK measures for topical ruxolitinib formulations are also under investigation in phase II and III trials in patients with AD (NCT03011892, NCT03745638, NCT03745651). When ruxolitinib was administered topically, at doses ranging from 0.15% to 1.5% BID, the resulting plasma concentrations followed a sublinear correlation to the dose [159]. Additional investigation is required to delineate the differences between topical versus oral ruxolitinib absorption and elimination measures, as indicated by the differences in bioavailability.

Tofacitinib is also predominantly metabolized by hepatic cytochrome enzymes and tofacitinib exposure may be increased upon co-administration of cytochrome inhibitors. Unmetabolized tofacitinib is eliminated by the kidneys and this process accounts for approximately 30% of overall tofacitinib elimination. Thus, adjusting tofacitinib dosing is necessary in patients with hepatic and/or renal impairment [152]. Unlike ruxolitinib, tofacitinib’s metabolites appear to be inactive [160].

Baricitinib is a substrate for the organic anion transporter (OAT)3, the multidrug and toxin extrusion protein (MATE)2-K, P-glycoprotein (P-gp), and breast cancer resistance protein (BCRP) and undergoes active renal tubular secretion [161]. PK modeling studies suggest that NSAIDS, such as ibuprofen and diclofenac, which have inhibitory activity against OAT3, are unlikely to impact baricitinib metabolism or activity. However, the OAT3 inhibitor, probenecid, is likely to impact baricitinib’s bioavailability [161].

A compassionate use study of baricitinib in pediatric patients with chronic atypical neutrophilic dermatosis lipodystrophy and elevated temperature (CANDLE) and Stimulator of interferon genes (STING)-associated vasculopathy with onset in infancy (SAVI) found that estimated glomerular filtration impacted baricitinib clearance (NCT01724580, NCT02974595) [162]. The authors assumed that, although not conclusive, the data supported prior findings that pediatric patients with renal impairments should be monitored closely when taking baricitinib [162]. Baricitinib is thus not recommended for patients with impaired renal function [163]. Conversely, the youngest and smallest (by weight) CANDLE and SAVI patients had the highest clearance rates by weight, indicating the potential need to compensate by increasing baricitinib dosing intervals and dosages in young patients [162].

Upadacitinib is also excreted by the kidneys and metabolized by hepatic cytochrome enzymes. Use of the cytochrome enzyme, CYP3A4, inhibitors or inducers is not recommended in patients taking upadacitinib, as these drugs have been demonstrated to significantly impact upadacitinib PK measures [152].

Filgotinib is metabolized by carboxylesterases to GS829845. GS829845 exposure has been determined to be 16–20 times that of filgotinib in both healthy volunteers and RA patients. Thus, the clinical effects of filgotinib may be the result of both the drug and GS829845 [156]. Filgotinib is renally excreted; renal impairment has been demonstrated to decrease filgotinib and GS829845 clearance. Surprisingly, despite the decrease in clearance rates and resulting increased exposure, filgotinib still appears to be well tolerated in patients with severe renal impairment [164].

Jakinib’s rapid absorption and clearance well position jakinibs to be dosed to achieve symptom alleviation while mitigating the AEs, perhaps better than existing treatments, such as corticosteroids. However, the quick half-lives of jakinibs bring into question how jakinibs achieve such efficacy. Pertinent to such questions, in a study to evaluate the PK and PD drivers of tofacitinib efficacy, investigators evaluated data from four phase II studies of tofacitinib-treated RA patients. They found that the average serum concentration of tofacitinib, rather than the minimum or maximum serum concentration, correlated with clinical efficacy [115]. Similarly, in PsA patients, the average serum concentration of upadacitinib was found to be significantly associated with the percentage of patients that achieved significant clinical improvements. As clinical efficacy correlated with upadacitinib average serum concentration, so too did the incidence of serious infections or decreases in hemoglobin [165]. These data support the notion that jakinibs are efficacious via partial inhibition of JAKs over the dosing period, despite an incomplete inhibition of downstream pathways. In comparison to biologics’ ‘narrow’ mechanism of more complete inhibition of single pathways, it becomes difficult to compare the relationship between selectivity, PKs, and efficacy of biologics and jakinibs.

## 5. Jakinib Discontinuation

Jakinibs have demonstrated success in alleviating symptoms and disease states for many patients, yet treatment discontinuation is not insignificant. In a report that evaluated a four-year period across RCTs for RA, encompassing over 18,000 patients, tofacitinib users were an average age of 56 years old, predominantly women, typically had more co-morbidities (including diabetes and hypertension), had previously been more compliant to prior prescribed treatment, and had used less methotrexate and more leflunomide and prednisone in prior treatments when compared to new bDMARD users. By the end of the studies surveyed, 57.3% of new tofacitinib users discontinued tofacitinib use, while 52.5% of new bDMARD users discontinued bDMARD use. Of those that discontinued treatment, tofacitinib users had a shorter treatment persistence compared to bDMARD users, with a mean of 0.81 and 1.02 years, respectively [162]. In a LTE study in RA patients, 45.2% and 50.7% of patients discontinued tofacitinib monotherapy and combination therapy, respectively, prior to extension completion (NCT00661661) [166]. Of those that discontinued treatment prematurely, 13.2% of monotherapy patients and 17.2% of combination patients discontinued due to AEs and 3.0% of monotherapy patients and 3.8% of combination patients discontinued due to poor clinical response. No other reasons for discontinuation were reported to be directly related to tofacitinib usage [166].

In a recent comparison of drug retention in RA patients taking baricitinib, tofacitinib, and sarilumab, an IL-6 receptor (IL-6R) antibody, significant differences were found in the gender ratio, disease duration, methotrexate and salazosulfapyridine use, and prior use of a jakinibs between the groups. Sarilumab and baricitinib users were observed to have a greater history of jakinib use than tofacitinib users. Reasons for discontinuation included lack of treatment effectiveness, toxic AEs, non-toxic reasoning, and remission. No significant differences were found in the patient-reported reasons for discontinuation between groups. The retention rates among the groups were also not significantly different; however, the hazard ratio for discontinuation due to toxic AEs was lower for baricitinib and tofacitinib than for sarilumab [167]. In a real-world study of RA patients taking baricitinib, only 66.27% continued baricitinib use past 1 year. Of those that discontinued, 64.28% discontinued due to a lack of efficacy and 28.57% due to AEs. Characterization of the group that discontinued within the first year found that 66.67% were women, the mean age was 49 years old, 85.71% were non-/inadequate responders to previous therapy, 42.68% were prescribed baricitinib as a monotherapy, 57.14% used baricitinib in combination with csDMARDs, and 9.52% used baricitinib in combination with glucocorticoids. Of the patients who discontinued after 1 year of baricitinib, 82.26% were women, the mean age was 61.52 years old, 83.87% were previous non-/inadequate responders to previous therapy, 42.86% used baricitinib as a monotherapy and 58.06% used baricitinib in combination with csDMARDs. The analysis reported that the factors associated with continued use of baricitinib, or prolonged treatment, persistence were older age and being a woman [168].

## 6. Conclusions

Jakinib development has a complex history that has followed and led to improved understandings of JAK signaling, cytokine biology, hyperinflammation, autoimmunity, cell homeostasis, hematopoiesis and the diseases that emerge when these pathways and processes are dysregulated. Despite the initial failing of the first use of a jakinib (tofacitinib) in patients with allograft rejection, jakinibs have redeemed themselves as promising treatments not only for transplant management, but for treating a heterogeneous group of hyperinflammatory and autoimmune diseases. The successes of jakinibs have resulted in a class of drugs that is rapidly expanding with increasing drug selectivity, organ specificity, and indications for treatment. This is most clearly evidenced by the recent EUAs for baricitinib and tofacitinib in the management and treatment of COVID-19 symptoms, despite their typical immunosuppressive effects and associated risk for viral infection. In addition to the FDA’s EUAs, the European Alliance of Associations for Rheumatology (EULAR) recently updated their guidelines for the treatment of COVID-19 to include tofacitinib or baricitinib. The 2021 EULAR guidelines now suggest tofacitinib or baricitinib (in combination with glucocorticoids) as a potential treatment for hospitalized COVID-19 patients requiring oxygen therapy, non-invasive ventilation, or high flow oxygen [169].

While jakinibs have demonstrated success in treating COVID-19, the immunosuppressive effect of jakinibs on patients’ ability to generate an immune response to COVID-19 vaccines has also been a concern. A study focused on RA and psoriasis patients taking tofacitinib, baricitinib, or upadacitinib that received SARS-CoV-2 vaccines BNT162b2 (Pfizer-BioNTech), CX-024414 (Moderna), CHAdOx1 (AstraZeneca), or Ad.26.COV2.S (Janssen). Overall vaccine response rates in jakinib-treated patients remained high (>80%) and similar to that of patients taking alternative immunosuppressants, methotrexate, and glucocorticoids [170]. The factors associated with patients who were non-responsive to mRNA COVID-19 vaccines were age and use of upadacitinib [170]. The study showed that in patients aged 65 and older or treated with upadacitinib, further assessment may be necessary during and after the vaccine course to determine the potential need for additional or familial vaccine doses to mitigate non-response to the COVID-19 mRNA vaccines. Further, the study also showed that jakinibs do not appear to negatively impact the immune response to mRNA COVID-19 vaccines.

Still, research to improve patient’s experiences while using jakinibs remains necessary to mitigate their side effects and associated risk factors. Improving our understanding of jakinib safety profiles, PKs, patient compliance, and discontinuation warrants extensive research as first-generation jakinibs continue to be used in new contexts and second-generation jakinibs are more commonly prescribed. Tofacitinib’s and baricitinib’s success in treating COVID-19 patients, potentially by mitigating the effects of CRS, demonstrate the potential to expand the use of jakinibs to treat other illnesses associated with CRS. Tocilizumab, an anti-IL-6R mAb, and anakinra, an IL-1 receptor (IL-1R) agonist, are under investigation to reduce CRS-mediated toxicities in chimeric antigen receptor T-cell (CAR-T) therapy recipients (NCT04359784, NCT04150913) [171]. Thus, jakinibs, which also mediate cytokine-receptor signaling, present a potential tool to reduce CRS reactions in patients treated with CAR-T therapies and be better titrated for effective treatment due to their short half-lives.

The short half-lives and quick elimination of jakinibs and active metabolites also position jakinibs as an enticing option to be applied to treat a variety of conditions beyond current applications. Importantly, steroids and jakinibs overlap in their usage in some diseases, including arthritis, AD and GVHD, although the use of jakinibs appears to exhibit fewer associated AEs than steroid-counterparts, due to steroids’ effects on non-immune systems and processes. Additionally, patients using glucocorticoids have been shown to develop glucocorticoid resistance in some cases [172]. Jakinibs, therefore, pose an enticing alternative to steroid use in disorders characterized by hyperimmune signaling. Once more, jakinibs may offer an additional advantage in terms of patient safety given that their short half-lives and quick elimination routes do not necessitate tapering following prolonged use, unlike many steroids that require tapering to avoid adrenal axis injury or insufficiency [173].

Oclacitinib has also been largely successful in the treatment of AD in canines. Extending the use of jakinibs to other veterinary diseases, including bovine mastitis, an inflammatory reaction of the udder tissue commonly associated with bacterial infection, could also prove to have significant economic impacts, as mastitis is the most costly and common disease among dairy cattle [174].

Observational studies and post-hoc analyses have also proposed that approximately 40% of RA patients experience major depressive disorder and/or generalized anxiety disorder and that IL-6 could potentially play a role in the pathogenesis of depression [175,176]. To investigate IL-6 as a potential drug target in the treatment of depression, the Insight study seeks to assess the role of IL-6 in depression pathogenesis and the mechanisms by which it may affect mood and cognition by utilizing the IL-6 inhibitor, tocilizumab, in depressive patients with and without low-grade inflammation [176]. As IL-6 signals via the JAK/STAT pathway, jakinibs may prove to be a useful tool in mood disorders, greatly widening the scope of jakinib indications.

Overall, as the number and applications of jakinibs continue to widen across a large spectrum of illnesses and species, this pharmaceutical class has the potential to effectively treat many difficult-to-manage diseases, further warranting continued investigation of jakinibs.

## Figures and Tables

**Table 1:** Summary of currently approved jakinibs, including JAK selectivity and indications.

JAK Inhibitor	Target	Indication	References
tofacitinib	JAK1, JAK3	RA, PsA, UC, adult and juvenile PsA (patients > 2)	[23]
	JAK2	juvenile idiopathic arthritis (patients > 2 years old)	
ruxolitinib	JAK1	PV, intermediate-high myelofibrosis, SR-GVHD	[22,24]
	JAK2	AD (topical cream)	
baricitinib	JAK1	RA ^1^, AD ^2^	[25,26]
	JAK2	COVID-19 ^3^	
upadacitinib	JAK1	RA, PsA, AS ^4^, moderate-severe AD ^4^	[27,28]
filgotinib	JAK1	RA ^4^, UC ^2^	[29]
abrocitinib	JAK1	AD ^5^	[30]
fedratinib	JAK2	intermediate-high risk	[31]
	FLT3	primary or secondary myelofibrosis	
peficitinib	pan-JAK	RA ^6^	[32]
delgocitinib	pan-JAK	AD ^7^	[33]
ocalacitinib	JAK1, JAK2	canine allergic dermatitis	[34]
	JAK3		

RA: rheumatoid arthritis, PsA: psoriatic arthritis, UC: ulcerative colitis, PV: polycythemia vera, SR-GVHD: steroid refractory-graft-versus-host-disease, AD: atopic dermatitis, AS: ankylosing spondyloarthritis. ^1^ 2 mg and 4 mg doses approved by the European Medicines Agency (EMA), only the 4 mg dose approved by the Food and Drug Administration (FDA). ^2^ Approved by the EMA, not approved by the FDA. ^3^ FDA Emergency Use Authorization for 4 mg baricitinib monotherapy and baricitinib in combination with remdesivir for treatment of hospitalized-COVID-19 patients over 2 years old requiring supplemental oxygen, ventilation or extracorporeal membrane oxygenation. ^4^ Approved by the EMA, not yet approved by the FDA. ^5^ Approved by the United Kingdom Medicines and Healthcare Products Regulatory Agency to treat severe AD in adults and adolescents 12 years and up, also granted FDA Priority Review and is Recommended for EMA Approval. ^6^ Approved in South Korea and Japan only. ^7^ Approved by the Japanese Pharmaceutical and Medical Devices Agency.

**Table 2:** Summary of jakinib trials.

JAK Inhibitor	Indication under Investigation	Trial
ruxolitinib	pediatric moderate-severe chronic GVHD	NCT03774082
	hemophagocytic lymphohistiocytosis	NCT04551131
	hypereosinophilic syndrome/primary eosinophilic disorders	NCT03801434
	inflammation in HIV patients	NCT02475655
	severe AA ^1^	NCT04518995, NCT04797650
tofacitinib	AS	NCT03502616
	SLE	NCT02535689
	cutaneous lupus	NCT03288324
	Sjogren’s syndrome	NCT04496960
	inflammatory eye disease	NCT03580343
	COVID-19	NCT04469114
baricitinib	SLE	NCT02708095, NCT03616912, NCT03616964, NCT03843125
	COVID-19	NCT04401579, NCT04640168, NCT04421027
peficitinib	RA	NCT01649999, NCT01711814, NCT01565655, NCT02308163, NCT02305849
	psoriasis	NCT01096862
	UC	NCT01959282
	impaired renal function	NCT02603497
delgocitinib	chronic hand eczema	NCT04871711, NCT04872101, NCT04949841
	inverse psoriasis	NCT02695940
	AD	NCT03725722, NCT03826901
upadacitinib	PsA	NCT03104374, NCT03104400, NCT03104374
	AS	NCT03178487
	AD ^2^	NCT03569293, NCT03607422, NCT03568318
	giant cell arteritis	NCT03725202
	Takayasu arteritis	NCT04161898
filgotinib	CD	NCT03077412, NCT02914561, NCT02914600
	PsA	NCT03101670
	AS	NCT03117270
abrocitinib	AD	NCT03349060, NCT03575871, NCT03720470
itacitinib	acute GVHD ^3^	NCT03139604
	asthma	NCT04129931
	bronchiolitis obliterans syndrome	NCT03978637
	MDS/MPN overlap syndrome	NCT04061421
SHR0302	CD	NCT03677648
	RA	NCT02892370, NCT02665910, NCT02423538
	AS	NCT04481139
	AA	NCT04346316
	AD	NCT04717310, NCT04162899
	UC	NCT03675477
	vitiligo	NCT04774809
deucravacitinib	psoriasis	NCT03624127, NCT03611751, NCT04167462, NCT03924427, NCT04036435
	PsA	NCT03881059
	CD	NCT03599622
	UC	NCT03934216
	SLE	NCT03252587
brepocitinib	psoriasis ^4^	NCT03850483
	AD ^1^	NCT03903822
	PsA	NCT03963401
	UC	NCT02958865
	CD	NCT03395184
	vitiligo	NCT03715829
	SLE	NCT03845517
	cicatricial alopecia	NCT05076006
	hidradenitis suppurativa	NCT04092452
	AA	NCT02974868
gusacitinib	AD	NCT03531957
	chronic hand eczema	NCT03728504
cerdulatinib	follicular lymphoma	NCT04021082
	T-cell lymphoma	NCT01994382
	vitiligo ^4^	NCT04103060
momelotinib	myelofibrosis	NCT01969838
	primary myelofibrosis, post-PV, post-ET myelofibrosis	NCT02101268
pacritinib	severe thrombocytopenia in myelofibrosis patients	NCT01773187
ritlecitinib	AA	NCT03732807
	vitiligo	NCT03715829
	UC	NCT02958865
	CD	NCT03395184
	AA	NCT04006457
izencitinib	UC	NCT03758443
	CD	NCT03635112
OST-122	UC	NCT04353791
TD-8236	asthma	NCT03652038, NCT04150341 ^5^
nezulcitinib	COVID-19-associated pulmonary disease	NCT04402866

^1^ Deuterated ruxolitinib, CTP-543. ^2^ As a monotherapy and in combination with topical corticosteroids. ^3^ Failed to meet primary endpoint. ^4^ Topical cream formulation. ^5^ Failed to meet primary endpoint. GVHD: graft-versus-host-disease; AD: atopic dermatitis; HIV: human immunodeficiency virus; AA: alopecia areata; AS: ankylosing spondyloarthritis; SLE: systemic lupus erythematosus; RA: rheumatoid arthritis; UC: ulcerative colitis; PsA: psoriatic arthritis; CD: Crohn’s disease; MDS/MPN: myelodysplastic/myeloproliferative; post-PV: post-polycythemia vera; post-ET: post-essential thrombocythemia.

**Table 3:** Summary of jakinib time to peak concentration and functional half-life.

JAK Inhibitor	Time to Peak Concentration	Half-Life (Hours)	References
ruxolitinib	<2 h	approximately 3 h	[151]
5 most common ruxolitinib metabolites	1.5–2 h	4.3–5.7 h	[150]
tofacitinib	0.5–1 h	3 h	[152]
baricitinib	0.75–1 h	5.9–7.4 h	[153]
upadacitinib	within 1 h ^1^; 2–3 h ^2^	4 h	[154,155]
filgotinib	0.5–5 h	5–6 h	[152]
GS829845	3–5 h	20–23 h	[156]

^1^ Immediate-release formula. ^2^ Extended-release formula.

## Data Availability

All data are contained in the main text of the article.
